# Prognosis and clinical issues of esophageal atresia in extremely low birth weight neonates: a case series

**DOI:** 10.1186/s12887-023-04237-1

**Published:** 2023-08-16

**Authors:** Masaki Horiike, Hitomi Mimura, Akiko Yokoi

**Affiliations:** 1 Department of Pediatric Surgery, Hyogo Prefectural Kobe Children’s Hospital, 650-0047 1-6-7, Minatojima Minamimachi, Chuo-Ku, Kobe Japan; 2 Department of Neonatal Medicine, Hyogo Prefectural Kobe Children’s Hospital, 650-0047 1-6-7, Minatojima Minamimachi, Chuo-Ku, Kobe Japan

**Keywords:** Esophageal atresia, Extremely low birth weight neonates, Clinical courses, Surgical procedures, Prognosis

## Abstract

**Background:**

Esophageal atresia (EA) in extremely low birth weight (ELBW) neonates is rare. This report aims to clarify EA’s clinical courses and prognosis in ELBW neonates and the clinical issues of long-term survival cases.

**Methods:**

A retrospective analysis was conducted for 8 neonates diagnosed with esophageal atresia. Medical records of ELBW EA neonates treated at our institution were reviewed to assess patient demographics, clinical courses, and outcomes. Transferred patient data was obtained from their local physicians through questionnaires.

**Results:**

EA in ELBW neonates were included in 8 of EA infants (7%). Fatal respiratory and cardiovascular complications of trisomy 18 and complications related to immaturity such as liver failure and pulmonary hypertension were associated with poor prognosis. During primary operations, gastrostomy and esophageal banding were performed together in 50% of the cases, while gastrostomy was performed alone in 25%. The esophageal anastomosis was not performed during any primary operation. All causes of death, except for 1 case, were due to non-surgical causes. A long-term survival case of 17 years postoperatively was included.

**Conclusion:**

Although ELBW EA has a poor prognosis, long-term survival is possible in some cases, so aggressive therapeutic intervention is considered essential. It is important to share information about the prognosis with parents and multidisciplinary specialists and to select an appropriate treatment strategy for each case.

## Background

Recent progress in neonatal medical care has improved the survival of extremely low birth weight (ELBW) neonates [1]. However, ELBW neonates are still independent predictive factors associated with in-hospital deaths of pediatric surgical patients in the NICU. In addition, postnatal growth failure in ELBW neonates is common and associated with worse neurodevelopmental outcomes [2–4]. Little is known about the clinical course and prognosis after medical and surgical intervention of esophageal atresia (EA) in ELBW neonates. While previous literature on EA in ELBW exists, most have focused on postoperative outcomes, whereas little has been written on long-term clinical courses [5–9].

Since the prognosis of ELBW has improved dramatically in recent years, the prognosis may also improve in EA in ELBW if appropriate treatment strategies are implemented.

We reviewed our experience managing this high-risk group of patients, focusing on clinical courses and medical conditions from the perinatal/perioperative period to home care to clarify EA's prognosis and clinical issues in ELBW.

## Method

This study was approved by the Research Ethics Board (registered no. R4-54). We performed an institutional-based retrospective analysis among ELBW (< 1000 g) EA neonates admitted to the NICU from August 1999 to December 2020 at Kobe Children’s Hospital. Medical records were reviewed to assess demographics such as birth weight, gestational age, sex, esophageal atresia classification, combined chromosomal abnormalities, cardiac anomalies, other associated anomalies, surgical procedures, hospital discharges, prognoses, causes of death, and lifespan.

Data on postoperative complications, in-home treatment (tracheotomy, home oxygen, ventilator, gastrostomy nutrition, parenteral nutrition), hospital visits, mental health development, motor function development, school life, and problems in daily life were also extracted from medical records. To collect data for transferred cases, we interviewed patients’ local physicians and families over the phone and sent questionnaires.

We defined the long-term survival cases (LT group) as patients with a lifespan of one year or more, and the short-term survival cases (ST group) as those with a lifespan of less than one year.

The cases in this study were categorized into the LT and ST group. Clinical findings, surgical procedures, and prognosis were compared between the two groups.

### Statistical analysis

The median and range of birth weight, gestational age, and lifespan were calculated using SPSS statistics ver. 27.

## Results

One hundred thirteen infants with EA (11 cases with type A, 1 with type B, and 101 cases with type C) were admitted to our institute over a 22-year period. 8 ELBW neonates with EA qualified for this case series (7%). Table 1 summarizes the clinical findings, surgical procedures, and prognoses of EA in ELBW neonates. The median and ranges (from minimum to max value) of birth weights, gestational ages, and lifespan was 702 (422–924) g, 29 (24–32) weeks, and 347 (0–5958) days, respectively. There were 2 cases of type A EA, and 6 cases of type C EA. Chromosome abnormalities were observed in 4 cases, all of which were trisomy 18.

**Table 1:** Clinical findings, surgical procedures and prognosis of esophageal atresia in extremely low birth weight neonates

									operations for esophageal atresia						
Gender	Birth weight	Gestational age	EA type	Chromosomal abnormality	Cardiac anomalies	PH	Cardiac operation	Other associated anomalies	Primary operation	Second operation	Third operation	Hospital discharge	Prognosis	Cause of death	Lifespan ( months)
M	862 g	27 weeks	A	—	PDA		PDAC (day6)	ー	GS (day6)	EPS (day103)	EEA (day264)	yes	dead	BPD-PH	51
F	604 g	24 weeks	C	—	PDA		-	CDA	LTEF (day5)	GS,DDA (day8)	EEA (day224)	yes	alive	-	198
F	754 g	29 weeks	C	trisomy 18	PDA,VSD,ASD	+	-	Myelomeningocele	GS, EB (day2)			yes	dead	UTI,sepsis	43
M	452 g	32 weeks	A	—	VSD, ASD	+	-	VATER association	GS (day4)			no	alive	-	13
M	650 g	29 weeks	C	trisomy 18	N.A		-	HL	—	—	—	no	dead	HL	0
F	836 g	30 weeks	C	trisomy 18	PDA, TGA, PS, VSD	+	-	ー	GS, EB (day3)			no	dead	CHF	0
M	924 g	31 weeks	C	trisomy 18	PDA, DORV, VSD, ASD	+	-	CDH	GS, EB (day1)			no	dead	PH	9
F	422 g	24 weeks	C	—	PDA, VSD		-	CDA	GS (day5)	ENS (day17)	LTEF (day59)	no	dead	LF	3

*PDA* patent ductus arteriosus, *TGA* transposition of the great arteries, *PS* pulmonary stenosis, *VSD* ventricular septal defect, *DORV* double-outlet right ventricle, *ASD* atrial septal defect, *CDA* congenital duodenal atresia, *HL* hypoplasia of lung, *CDH* congenital diaphragmatic hernia, *GS* gastrostomy, *EB* esophageal banding, *PDAC* patent ductus arteriosus clipping, *EPS* esophagostomy, *ENS* enterostomy, *EEA* esophageal anastomosis, *LTEF* ligation of trachoesophageal fistula, *DDA* duodeno-duodeno anastomosis, *PH* pulmonary hypertension, *CHF* congestion heart failure, *UTI* urinary tract infection, *LF* liver failure, *BPD-PH* Pulmonary hypertension associated with Bronchopulmonary dysplasia, *N. A* not assessed

Cardiac anomalies were observed in 7 cases, with 2 cases having major anomalies and 5 cases having minor anomalies. The major anomalies were Transposition of the great arteries and Double-outlet right ventricle, both of which involved merging PS and VSD or ASD and VSD simultaneously. The minor anomalies were associated with PDA, ASD, and VSD, either alone or in combination.

Associated anomalies included 2 cases of congenital duodenal atresia (CDA), 1 case each of hypoplasia of the lung, congenital diaphragmatic hernia (CDH), myelomeningocele, and VATER association. During the primary operation, three neonates underwent gastrostomy as well as esophageal banding which is a procedure used for type C esophageal atresia with tracheoesophageal fistula (TEF) to prevent gastric acid from flowing back into the lungs through the TEF, 3 underwent gastrostomy alone, 1 underwent ligation of tracheoesophageal fistula, and one never underwent any surgery due to his short lifespan. We performed a second operation in 3 cases: esophagostomy in case number 1, gastrostomy and duodeno-duodeno anastomosis in case number 2, and an enterostomy in case number 8. The same 3 cases also underwent a third operation: esophageal anastomosis and pyloroplasty in case number 1, a tracheostomy in case number 2, and ligation of tracheoesophageal fistula in case number 8.

The postoperative course of each case classified into LT and ST groups is listed in Table 2. There was no apparent difference between the two groups in sex, birth weight, gestation age, and pulmonary hypertension (PH) derived from Cardiac anomalies.

**Table 2:** Comparison between LT and ST groups for clinical findings, surgical procedures and prognosis

	LT groups (*n* = 4)	ST groups (*n* = 4)
Gender (M/F)	2/2	2/2
Birth weight (median (range) g)	679 (401–904)	743 (412–924)
Gestation age (weeks)	28 (24–32)	29.5 (24–31)
Lifespan (days)	1419 (413–5958)	58.5 (0–282)
chromosomal abnormality		
18trisomy	1/4 (25%)	3/4 (75%)
another	0	0
PH derived from Cardiac anomalies	2	2
PAB	0	0
Another associated anomalies		
VATER association	1	0
Myelomeningocele	1	0
CDA	1	1
CDH	0	1
HL	0	1
Primary operation		
LTEF	1	0
GS + EB	1	2
GS	2	1
none	0	1
Hospital discharge	3/4 (75%)	0 (0%)
Prognosis ( alive / dead)	2/2	0/4

*LT* long-term survival, *ST* short-term survival, *PH* pulmonary hypertension, *PAB* pulmonary artery banding, *CDA* congenital duodenal atresia, *HL* hypoplasia of lung, *LTEF* ligation of trachoesophageal fistula, *GS* + *EB* gastrostomy + esophageal banding

Trisomy 18 was found most often in the ST group. In addition, fatal lung hypoplasia was found in the ST group. In the LT group, 75% were discharged, and 50% were long-term survivors.

The postoperative conditions and required medical care for each case are listed in Table 3. Case number 5 died shortly after birth due to hypoplasia of the lung and was not operated on, so it was excluded from consideration of postoperative status and medical care status. All 7 cases required oxygen, and 6 cases were mechanically ventilated. Tracheotomy was performed in 4 cases. Gastrostomy nutrition was performed in 5 of the 7 cases, and enterostomy was administered in 1. Parenteral nutrition was performed in 2 of the 7 cases. Postoperative complications were gastroesophageal reflux (GER) and aspiration pneumonia in 3 cases, and enterostomy dysfunction, enterostomy subcutaneous abscess, intestinal failure associated liver disease (IFALD), and catheter-related bloodstream infection (CRBSI) in 1 case. Case numbers 1–3 were discharged from the hospital, followed by regular check-ups. However, case number 3 had to make frequent outpatient visits due to recurrent urinary tract infections (UTIs) caused by Myelomeningocele. Mental and motor retardation was observed in all five evaluable cases. As for growth, severe growth failure was observed in all 7 cases. Concerning problems in daily life, dysphagia was observed in 2 cases who underwent esophageal anastomosis, recurrent UTIs were observed in 1 case with spinal meningocele, and 1 case with pulmonary hypertension (PH) required constant use of nitric oxide (NO).

**Table 3:** Postoperative conditions and requried medical cares of the cases that performed surgical intervention in their duration of survival

case	oxygen therapy	tracheostomy	use of a ventilator	gastrostomy nutrition	parenteral nutrition	post operative complication	annual hospital care	reason for hospital visit	mental retardation	motor retardation	physical development (SD)		school attendance	challenges in daily life
											height	weight		
1	yes	no	no	no	no	GER,aspiration penumonia	4 times or less	regular follow up	yes	yes	-2.1	-1.9	pre-school child	mild difficulty in swallowing,respiratory tract susceptibility,
2	yes	yes	yes	yes	no	GER,aspiration penumonia	3 times or less	regular follow up	yes	yes	-4.1	-4.2	handicapped children's class	mild difficulty in swallowing
3	yes	yes	yes	yes	no	-	12 times or more	regular follow up, recurrent UTI	yes	yes	-13	-23	pre-school child	recurrent UTI
4	yes	yes	yes	yes	no	GER,aspiration penumonia	long-term hospitalization	long-term hospitalization	yes	yes	-9.5	-10.3	pre-school child	regular use of NO due to PH
6	yes	intubation management	yes	yes	yes	-	-	-	not applicable	not applicable	-7.3	-5.6	pre-school child	
7	yes	yes	yes	yes	no	-	long-term hospitalization	long-term hospitalization	not applicable	not applicable	-5.4	-7.1	pre-school child	
8	yes	intubation management	yes	no (enterostomy nutrition)	yes	ED,ESA,IFALD,CRBSI	long-term hospitalization	long-term hospitalization	not applicable	not applicable	-11	-4.7	pre-school child	

*GER* gastroesophageal reflux, *UTI* urinary tract infection, *ED* enterostomy dysfunction, *ESA* enterostomy subcutaneous abscess, *IFALD* intestinal failure associated liver disease, *CRBSI* catheter related bloodstream infection, *NO* nitric oxide, *PH* pulmonary hypertension

## Discussion

We analyzed the clinical course and prognosis of 8 EA cases in ELBW neonates born after 24 weeks of gestation and treated at our hospital over a 22-year.

The management of neonatal intensive care units has undergone significant enhancements. Alongside advancements in ventilators, the adoption of appropriate management techniques has been widespread due to the nationwide accumulation of detailed respiratory and circulatory management methods for ELBW infants. In terms of surgery, it is important to have a surgical strategy to establish enteral nutrition from early birth and a strategy to proceed with surgery step by step according to the patient's general condition for esophageal atresia. As a result, the survival rate and quality of life for such infants have witnessed remarkable improvements.

The overall survival rate of ELBW neonates born after 24 weeks of gestation in Japan is 65.8–95.2% [10–12]. In comparison, the survival rate for the neonates in this case series was 37.5%. This result is consistent with previous literature [5, 9] suggesting that while ELBW lowers the survival rate, EA and associated anomalies are significant prognostic factors that further lower the survival rate.

Table 2 shows a high prevalence of trisomy 18 among the case series in this study. The prognosis for trisomy 18 was reported to be 17–27% for the one-year survival rate [13–15]. Tamaki et al. [16] reported that 1-year, 3-year, and 5-year survival rates of trisomy 18 neonates admitted to the NICU from 2013 to 2017 were 59.3, 44.4, and 33.3%, respectively. This report shows that while aggressive surgical intervention increased the survival rate in cases with trisomy 18, it was not enough to sustain long-term survival. These findings further demonstrate that the complication of trisomy 18 is a poor prognostic factor. Furthermore, trisomy 18 is known to be a common complication with cardiac anomalies at a high rate.

Cardiac anomalies leading to PH were observed in 2 cases in both groups, managed without pulmonary arterial banding to avoid the risk of high surgical invasiveness. The cause of death for two patients with a trisomy 18 complication was PH and CHF. The low number of cases in this series did not allow us to assess the impact of cardiac anomalies; therefore, we could not prove causation between cardiac anomalies and a poor prognosis.

Lopez PJ, et al. [17] mentioned that the number of infants weighing less than 1500 g has increased. Antenatal diagnoses of significant congenital anomalies which affect prognosis, such as cardiac anomalies, are also increasing in accuracy. Accurate information on these anomalies could better facilitate parents' decision-making when considering whether to continue with the pregnancy. Pediatric surgeons should recognize that many neonates may have poor prognoses due to factors other than surgical complications. Therefore, focusing on providing parents and caregivers with an accurate prognosis is essential.

Only 2 of the 7 cases in this study required parenteral nutrition. This was the result of our strong awareness of the importance of establishing enteral nutrition for EA cases in ELBW neonates, and the establishment of a gastrostomy early after birth and the start of enteral nutrition.

The surgical approach to EA in ELBW neonates should be individualized to find the best intervention for esophageal anastomosis. Regarding indication for ligation of tracheoesophageal fistula, we selected cases with high respiratory and circulatory dynamic stability. Seitz. et al. [18] reported that primary repair of EA and ligation of tracheoesophageal fistula was achievable with ELBW neonates. Conversely, Petrosyan et al. [19] reported that staged esophageal repair for EA in VLBW neonates results in a lower rate of anastomotic complications and overall morbidity than primary repair. There were no postoperative complications after performing gastrostomy with esophageal banding (4 cases), gastrostomy (1 case), and ligation of tracheoesophageal fistula without primary repair for EA (1 case). We performed ligation of tracheoesophageal fistula in 1 case with stable respiratory and circulatory dynamics without chromosomal abnormalities or severe cardiac malformations, and the course has been favorable thus far.

Furthermore, esophageal anastomosis was performed during the third and fourth operations for 2 cases with a stable clinical course. One has achieved long-term survival maintained with nutritional management through oral intake and gastrostomy. Appropriate surgical procedure selection for each case can ensure QOL without causing serious surgery-related complications. Depending on the case, the patient can shift to home management and achieve a longer life span.

The LT group had a median survival age of approximately 4 years. However, one patient has survived for 17 years thus far; in this case, respiration was managed through a tracheostomy cannula and nutrition through a combination of oral intake and gastrostomy nutrition. Growth and psychomotor retardation were evident. The patient has had difficulty living independently due to the required suction of phlegm and gastrostomy nutrition management; however, she has been able to hold a few part-time jobs.

Our study was retrospective, had a small sample size, and was partially biased by phone calls and questionnaires, and we must limit our conclusions to generalizations. However, we hope these results will be shared with pediatric surgeons and multidisciplinary specialists to improve and lead to better education and clinical attitudes when caring for EA in ELBW neonates.

## Conclusion

This study's cases had a high mortality rate where intensive medical interventions were required. Instead of surgical complications, Trisomy 18, severe cardiac anomalies, and lung hypoplasia seemed to determine the prognosis. There was one case with growth and psychomotor retardation who survived with ventilator management for more than 17 years thus far. The patient has had difficulty living independently due to the required suction of phlegm and gastrostomy nutrition management; however, she has been able to hold a few part-time jobs.

ELBW EA who do not have fatal respiratory and cardiovascular complications of trisomy 18 complications and immaturity-related complications such as liver failure and pulmonary hypertension have the potential for long-term survival. Therefore, we should provide information about the expected clinical course and prognosis on long-term surviving patients and proceed with aggressive systemic management and surgical treatment.

## Data Availability

The datasets analyzed during the current study are available from the corresponding author on reasonable request.

## Abbreviations

EA: Esophageal atresia
ELBW: Extremely low birth weight
PDA: Patent ductus arteriosus
VSD: Ventricular septal defect
ASD: Atrial septal defect
PS: Pulmonary stenosis
PDAC: Patent ductus arteriosus clipping
CDA: Congenital duodenal atresia
CDH: Congenital diaphragmatic hernia
BPD-PH: Pulmonary hypertension associated with Bronchopulmonary dysplasia
ES: Enterostomy
PH: Pulmonary hypertension
CHF: Congestion heart failure
UTI: Urinary tract infection
LF: Liver failure
LT: Long-term survival
ST: Short-term survival
GER: Gastroesophageal reflux
IFALD: Intestinal failure associated liver disease
CRBSI: Catheter-related bloodstream infection
NO: Nitric oxide
